# Sea buckthorn pulp and seed oils ameliorate lipid metabolism disorders and modulate gut microbiota in C57BL/6J mice on high-fat diet

**DOI:** 10.3389/fnut.2022.1067813

**Published:** 2022-12-08

**Authors:** Zhen Wang, Shengmin Zhou, Yuanrong Jiang

**Affiliations:** ^1^Wilmar (Shanghai) Biotechnology Research & Development Center Co., Ltd., Shanghai, China; ^2^School of Public Health (Shenzhen), Shenzhen Campus of Sun Yat-sen University, Sun Yat-sen University, Shenzhen, China

**Keywords:** sea buckthorn pulp oil (SBPO), sea buckthorn seed oil (SBSO), nonalcoholic fatty liver disease (NAFLD), gut microbiota, high-fat diet

## Abstract

**Introduction:**

Non-alcoholic fatty liver diseases (NAFLD), along with the complications of obesity and dyslipidemia, are worldwide lipid metabolism disorders. Recent evidence showed that NAFLD could be ameliorated by diet and lifestyles by attenuating gut microbiota dysbiosis via the gut–liver axis. Sea buckthorn oils, including sea buckthorn pulp oil (SBPO) and sea buckthorn seed oil (SBSO), were investigated in this study for their beneficial effects on gut–liver axis in C57BL/6J mice on a high-fat diet.

**Methods:**

Sixty of male C57BL/6J mice were assigned into five groups, fed with low-fat diet containing soybean oil (SO), high-fat diet comprising lard oil (LO), peanut oil (PO), SBSO or SBPO, respectively, for 12 weeks. Serum and hepatic biochemical analysis, liver and perirenal fat histological analysis, and fecal 16S rRNA gene sequencing were conducted to reflect the influence of five diets on gut-liver axis.

**Results:**

Dietary SBPO reduced visceral fat accumulation, adipose cell size, serum and hepatic triglyceride, LDL-C levels, and hepatic cell damage score; increased gut microbiota diversity with a higher abundance of *Lactobacillus*, *Roseburia*, and *Oscillibacter* compared with PO. SBSO showed equal or weaker effects compared to SBPO.

**Conclusion:**

This study demonstrates that dietary SBPO has the potential to ameliorate NAFLD and related metabolic disorders, like obesity and dyslipidemia, by modulating gut microbiota.

## Introduction

Non-alcoholic fatty liver disease (NAFLD), which was internationally renamed as metabolic dysfunction-associated fatty liver disease (MAFLD) in 2020 to better reflect the inclusion of metabolic abnormalities diagnostic criteria (1, 2), is a global liver disorder prevalent in approximately 15% of the Chinese population and even higher at 25% of the worldwide population (3, 4). MAFLD, in the text referred to as NAFLD, and the more progressive stage of metabolic dysfunction-associated steatohepatitis (MASH, in the text referred as NASH) are common lipid metabolism disorders starting from liver steatosis, and with complications of obesity, dyslipidemia, and type 2 diabetes mellitus (T2DM) (5–7). However, the pathogenesis of NAFLD is still under investigation (6). In recent years, human gut microbiota dysbiosis tends to be regarded as one of the linkages between obesity and metabolic diseases including NAFLD (5). The term “gut–liver axis” was proposed to describe the association and interdependence between the gut and liver from the embryo period (8). By gut microbiota transplanting, mice treated with NAFLD human microbiota showed higher levels of body weight, liver triglyceride, and plasma low-density lipoprotein cholesterol than mice with healthy human microbiota, verifying the crucial role of gut microbiota in the development of liver steatosis and obesity (9).

Important factors affecting the gut–liver axis include diet and lifestyle interventions, which are potential targets for the prevention and/or treatment of NAFLD (8). High-fat diet (HFD) rich in saturated fat sources, such as lard and palm oil, could disturb the gut–liver axis by inducing liver steatosis, stimulating colonocyte dysfunction, suppressing gut microbial diversity, and modulating the relative abundance of microbes (4, 8, 10). Numerous dietary sources have been explored for attenuating HFD-induced NAFLD, obesity, and gut microbiota dysbiosis in C57BL/6J mice model, for instance, polyphenol from oil seeds (3), flavonoids from whole-grain oat (11), ascorbic acid (12), and so on. Previous studies on dietary oil using the hypercholesterolemia hamster model also found that wild melon seed oil could reduce plasma cholesterol at least in part by modulating gut microbiota; and the repeated reuse of frying corn and lard oils could increase the plasma triacylglycerol and reduce the richness and diversity of gut microbiota at the same time (13, 14). Besides the aforementioned studies, it barely can be seen the investigation of the impact of oil selection on HFD-induced metabolic disorders.

Sea buckthorn (*Hippophae rhamnoides* L.) is one of the valuable plants widely cultivated in mountainous areas of Asia including China, India, Mongolia, and Russia, as well as North Western Europe and North America (15, 16). It has been recognized as herbal medicine in ancient China and India, and recently emerged as a functional food due to its health applications in cardiovascular, gastrointestinal, and skin diseases (16, 17). Particularly, sea buckthorn oil is one of the major products commercialized in the market, featured by lipophilic bioactive compounds such as polyphenols, phytosterols, unsaturated fatty acids, and carotenoids (15, 18). Sea buckthorn oil could be classified into sea buckthorn seed oil (SBSO) and sea buckthorn pulp oil (SBPO) based on the extraction positions (16, 19, 20). Both SBSO and SBPO are rich in tocopherols, especially α-tocopherol, and phytosterols, especially β-sitosterol (15, 19). SBPO possesses 20–45% of the n-7 fatty acid palmitoleic acid (C16:1n-7) in fatty acid composition, which is uncommon in plant sources and barely found in SBSO (15, 18, 21). While SBSO has more polyunsaturated fatty acids including around 30% each of linoleic acid (C18:2n-6) and linolenic acid (C18:3n-3), contributing to a balanced n-6/n-3 fatty acid profile with the ratio close to 1:1 in some species (15, 18, 21). Moreover, SBPO especially consists of concentrated carotenoid content, which is an important characteristic of SBPO in commercial trade (15).

The extraordinary abundance of bioactive compounds presented in SBSO and SBPO attracted us to pay attention to their potential health benefits. Palmitoleic acid (C16:1n-7) is important for skin and epithelial tissues including gastric mucosa and the inside of the eye; in the past, SBPO was investigated for the application in relieving skin burns, mucosa lesions, and dye eye syndrome (15, 18). Atopic dermatitis as a skin disease was significantly ameliorated by dietary SBPO but not SBSO (22). Both SBSO and SBPO showed anti-inflammatory activity and therefore have an influence on metabolic diseases, mainly attributed to the existence of key tocopherols, phytosterols, and carotenoids (23). Both SBSO and SBPO improved dyslipidemia (24, 25) and displayed anti-atherogenic effects (26, 27). Dietary SBSO from 0.26 to 2.6 mg/kg showed hepatoprotective properties by reducing liver lipid peroxidation, serum triglyceride, and serum cholesterol, indicating the potential of SBSO in the prevention and treatment of liver diseases (19, 28). Moreover, SBSO modulated gut microbiota in addition to reducing blood cholesterol in hamsters with hypercholesterolemia (25). Sea buckthorn freeze-dried powder also showed a strong linkage between gut microbiota alteration and HFD-induced obesity and associated lipid metabolism disorders (29). However, to the best of our knowledge, no one revealed the influence of dietary SBPO on the gut–liver axis, the connection between gut microbiota composition and liver disorders, and relevant metabolic diseases.

Therefore, in the present study, we investigated the beneficial effect of SBPO and SBSO on the gut–liver axis by evaluating the indicators of lipid metabolism disorders, that is, fat accumulation, dyslipidemia, and liver steatosis, as well as gut microbiota dysbiosis induced by high-fat diet in C57BL/6J mice. The comparisons were made between SBPO and SBSO because of their different bioactive compound profiles. Furthermore, both SBPO and SBSO were also compared to other fat contents commonly can be seen in the daily diet and especially in HFD, such as animal fat lard oil (LO) and plant oil peanut oil (PO), to see if SBPO and SBSO had potential to ameliorate metabolic disorders induced by HFD. A low-fat diet (LFD) containing soybean oil (SO) was used as the positive reference in this study.

## Materials and methods

### Materials

Refined SO, LO, and refined PO were kindly provided by Wilmar Biotechnology Research and Development Center Co., Ltd (Shanghai, China). SBSO and SBPO were commercial products (Qinghai Kangpu Biotechnology Co., Ltd, Qinghai, China) purchased from local market.

### Oil composition analysis

The fatty acid composition of tested oils was quantified on an Agilent gas chromatography (GC)–Flame Ionization Detector (FID) according to the AOCS Official Method Ce 1f-96 as described elsewhere (30). The tocopherol contents (α-, β-, γ-, δ-tocopherols) of tested oils were analyzed according to AOCS Official Method Ce 8-89, on an Agilent high-performance liquid chromatography (HPLC) system with a UV detector (292 nm) and a silica column (4.6 mm × 250 mm, 5 mm, Waters Sepherisorb Silica) (31). Besides, the measurements of phytosterols, squalene, and β-carotene levels were described elsewhere (31).

### Animal diets

Five diets, that is, a low-fat diet (LFD, 10% kcal fat) comprising 4% (w/w) of refined SO (L-SO), four parallel high-fat diets (HFD, 45% kcal fat) comprising 23.6% (w/w) of LO (H-LO), refined PO (H-PO), SBSO (H-SBSO), and SBPO (H-SBPO), respectively, were used in the study (Supplementary Table 1). They were manufactured by Opensource Animal Diets Co. Ltd (Changzhou, China) as pellets, visually differentiated by color, and frozen at −20°C until use.

### Animal study design

The animal study procedures were approved by the Shanghai Medicine Industry Research Institute (License Number, SYXK(Hu) 2014-0018), and animals were bred following the Chinese National Standard GB14925-2010. In brief, SPF-grade C57BL/6J mice were obtained from the Shanghai Jiesijie Laboratory Animal Co., Ltd (Shanghai, China) and grew at 12-h light–dark cycles, with feed and water *ad libitum*.

After 5 days of acclimatization period, 60 healthy male C57BL/6J mice at 19 ± 0.1 g were randomly assigned into five groups (*n* = 12) and fed with low-fat diet L-SO along with high-fat diet H-LO, H-PO, H-SBSO, H-SBPO, respectively, for 12 weeks. The high-fat diets containing 45 kcal% fat were supposed to induce the development of lipid metabolism disorders in C57BL/6J mice after a 12-week treatment period (12). The low-fat diet containing 10 kcal% fat was used as a positive reference to the HFD (12). Feed was provided and their intake was recorded two times a week. Mice were weighed one time a week. After fasting overnight, all mice were anesthetized with 50 mg/kg of pentobarbital sodium through intraperitoneal injection. Blood samples were collected by an intraorbital puncture. Then, the mice were euthanized by cervical dislocation, and liver, perirenal fat, epididymal fat, and feces from cecal contents were carefully collected, weighed, and partially stored at −80°C until analysis. Part of the liver and perirenal fat were fixed with 10% neutral buffered formalin for histological analysis.

### Biochemical analysis

The total triacylglycerol (TG), total cholesterol (TC), low-density lipoprotein cholesterol (LDL-C), and high-density lipoprotein cholesterol (HDL-C) were measured for serum and supernatant of the homogenized liver following the instructions of enzymatic assay kits (Nanjing Jiancheng, Nanjing, China), using Hitachi 7080 automatic biochemical analyzer (Hitachi, Ltd., Tokyo, Japan) and Thermo Scientific Variskan Flash microplate reader (Thermo Fisher Scientific, MA, United States).

### Histological analysis

Part of the liver and right perirenal fat fixed overnight in 10% neutral buffered formalin were dehydrated and sectioned at 5 μm, stained with diluted hematoxylin and eosin (H&E) followed by photographing (magnification of 400×) on a Nikon optical microscope (NIKON Eclipse ci, Minato, Japan). Histopathologic lesions of the liver sections were scored based on the criteria reported previously (32). Counts for the adipose cell numbers were performed by Image-pro plus 6.0 (Media Cybernetics, Inc., MD, United States) within the unit length on perirenal fat section images (magnification of 200×). The average diameter was calculated to represent the size of perirenal adipose cells.

### Fecal DNA extraction, PCR amplification, and sequencing

The 16S rRNA gene sequencing was performed on collected feces samples at the end of the experiment. Fecal DNA extraction, PCR amplification, and sequencing were similar to previous work (33) with minor modifications. In brief, bacterial genome DNA was extracted from 60 cecal content samples according to the manufacturer’s instructions of InviMag Stool DNA Kit/Kfml (STRATEC Molecular, Berlin, Germany). The concentration of extracted DNA was measured on Nano-Drop 2000 spectrophotometer (Nano-Drop Technologies, Wilmington, DE, United States). The extracted DNA samples were stored at −20°C for further analysis. Then, the bacterial 16S rRNA gene was amplified in a PCR system (ABI GeneAmp1 9700, Carlsbad, CA, USA) with the following procedure: Denaturing step at 95°C for 3 min and 30 cycles of 95°C for 30 s, annealing at 55°C for 30 s, and extension at 72°C for 45 s, followed by final annealing extension step at 72°C for 10 min using the primers 338F (5′-ACTCCTACGGGAGGCAGCAG-3′) and 806R (5′-GGACTACHVGGGTWTCTAAT-3′). The PCR reactions were performed in triplicate in a 20-μl mixture containing 4 μl of 5 × FastPfu buffer, 2 μl of 2.5 mM dNTPs, 0.8 μl of forward primer (5 μM), 0.8 μl of reverse primer (5 μM), 0.4 μl of FastPfu polymerase (TransGen, China), and 10 ng of template DNA. PCR products were visualized on a 2% agarose gel, purified using an AxyPrep DNA Gel Extraction Kit (Axygen Biosciences, Union City, CA, USA), and quantified with QuantiFluorTM-ST (Promega, USA). Pyrosequencing was performed on a MiSeq platform (Illumina, San Diego, CA, USA) at Majorbio Bio-pharm Technology Co., Ltd (Shanghai, China).

### Bioinformatic analysis

Sequencing data were analyzed using the Majorbio Bioinformatic Platform.^1^ Operational taxonomy units (OTUs) at a 97% similarity level were achieved with high-quality sequencing reads after removing low-quality sequences, pyrosequencing errors, and chimera. α-Diversity indices (Chao and Shannon) were performed using the MOTHUR software 1.30.2. β-Diversity distance calculation and BLASTs of taxonomic classification from phylum and genus level were performed using Qiime 1.9.1. Principal coordinates analysis (PCoA) was performed to provide an overview of gut microbial dynamics.

### Statistical analysis

Variables for oil fatty acid and micronutrient compositions (*n* = 3), along with mice body weight, daily intake, tissue weight, serum and liver biochemistry, liver and perirenal fat histopathological score, cecal content bacterial richness, and diversity indexes (*n* = 10–12) were expressed as mean ± standard deviation (SD). Significance between groups was analyzed by one-way analysis of variance (ANOVA) with *pos -hoc* Tukey’s test in OriginPro 8.5.1 Software (OriginLab Corporation, MA, United States, *p* < 0.05).

## Results

### Composition of fatty acid and micronutrients in tested oils

The fatty acid composition of the five tested oils is summarized in Table 1. Although from the same sea buckthorn species, SBSO and SBPO have distinctive fatty acid profiles. SBPO has much higher SFA and MUFA concentrations than other plant oils, that is, SO, PO, and SBSO, resembling the animal fat LO. Specifically, SBPO is featured by palmitic acid C16:0 and palmitoleic acid C16:1n-7, each accounting for approximately 31% of total fatty acids. A high abundance of palmitoleic acid C16:1n-7 is a characteristic of SBPO and it is attributable to various beneficial effects of SBPO (15). On the contrary, SBSO is high in PUFA, possessing around 35% of linoleic acid C18:2n-6 and 28% of α-linolenic acid C18:3 in profile. SBSO has a more balanced n-6/n-3 ratio (1.3:1) than SO (7.8:1), LO (12.8:1), and PO (37.3:1). The SBPO and SBSO fatty acid compositions are in line with previous studies (15, 21).

**TABLE 1:** Fatty acid and micronutrient compositions of tested oils*.

	SO	LO	PO	SBSO	SBPO
**FA (%)**					
C12:0	ND^b^	0.077 ± 0.001^a^	ND^b^	ND^b^	ND^b^
C14:0	0.046 ± 0.040^c^	1.331 ± 0.015^a^	ND^c^	0.050 ± 0.043^c^	0.594 ± 0.006^b^
C14:1	ND^b^	ND^b^	ND^b^	ND^b^	0.102 ± 0.002^a^
C15:0	ND^b^	ND^b^	ND^b^	ND^b^	0.195 ± 0.002^a^
C16:0	10.732 ± 0.012^c^	24.707 ± 0.205^b^	9.913 ± 0.031^d^	8.348 ± 0.037^e^	31.622 ± 0.216^a^
C16:1n-7	0.072 ± 0.010^c^	1.850 ± 0.023^b^	0.071 ± 0.007^c^	0.247 ± 0.008^c^	31.191 ± 0.185^a^
C17:0	0.088 ± 0.004^c^	0.224 ± 0.014^a^	0.059 ± 0.012^d^	0.043 ± 0.006^d^	0.161 ± 0.010^b^
C17:1	0.046 ± 0.005^c^	0.144 ± 0.013^b^	0.025 ± 0.008^d^	ND^e^	0.187 ± 0.001^a^
C18:0	3.976 ± 0.006^b^	14.340 ± 0.228^a^	3.590 ± 0.011^c^	2.789 ± 0.047^d^	1.015 ± 0.012^e^
C18:1	22.342 ± 0.089^e^	41.450 ± 0.598^b^	49.024 ± 0.055^a^	24.033 ± 0.049^d^	27.438 ± 0.412^c^
C18:2n-6	53.944 ± 0.027^a^	13.369 ± 0.251^d^	31.140 ± 0.090^c^	35.472 ± 0.036^b^	5.358 ± 0.028^e^
C18:3n-3	6.923 ± 0.039^b^	1.046 ± 0.118^d^	0.834 ± 0.020^e^	27.915 ± 0.066^a^	1.763 ± 0.049^c^
C20:0	0.321 ± 0.003^b^	0.217 ± 0.026^c^	1.215 ± 0.005^a^	0.312 ± 0.003^b^	0.322 ± 0.005^b^
C20:2	ND^b^	0.577 ± 0.005^a^	ND^b^	ND^b^	ND^b^
C22:0	0.349 ± 0.002^b^	ND^c^	2.330 ± 0.011^a^	ND^c^	ND^c^
C24:0	ND^b^	ND^b^	1.061 ± 0.005^a^	ND^b^	ND^b^
Trans FA¶	1.160 ± 0.044^a^	0.668 ± 0.048^b^	0.739 ± 0.026^b^	0.791 ± 0.109^c^	0.051 ± 0.003^c^
SFA	15.513 ± 0.042^d^	40.897 ± 0.489^a^	18.167 ± 0.070^c^	11.541 ± 0.111^e^	33.909 ± 0.216^b^
MUFA	22.461 ± 0.078^e^	43.443 ± 0.564^c^	49.121 ± 0.065^b^	24.280 ± 0.057^d^	58.918 ± 0.238^a^
PUFA	60.867 ± 0.042^b^	14.992 ± 0.138^d^	31.974 ± 0.109^c^	63.387 ± 0.032^a^	7.122 ± 0.028^e^
n-6/n-3#	7.8:1	12.8:1	37.3:1	1.3:1	3.0:1
**Micronutrients (mg/kg)**					
Total tocopherols	1,230.00 ± 5.29^c^	ND^e^	309.33 ± 6.51^d^	2,806.33 ± 14.22^a^	1,834.67 ± 91.82^b^
α-Tocopherol	120.13 ± 1.06^d^	ND^e^	204.47 ± 2.83^c^	1,179.20 ± 6.35^b^	1,621.23 ± 4.69^a^
β-Tocopherol	ND^b^	ND^b^	ND^b^	70.37 ± 6.64^a^	120.90 ± 57.44^a^
γ-Tocopherol	824.03 ± 3.23^b^	ND^e^	105.03 ± 4.32^c^	1,279.90 ± 8.42^a^	92.47 ± 39.98^d^
δ-Tocopherol	285.07 ± 0.91^a^	ND^c^	ND^c^	277.00 ± 3.96^b^	ND^c^
Total phytosterols	3,508.32 ± 28.59^c^	ND^e^	2,953.05 ± 50.27^d^	9,809.67 ± 31.68^b^	11,233.66 ± 62.99^a^
Campesterol	642.25 ± 2.33^b^	ND^e^	380.69 ± 10.08^c^	656.57 ± 0.94^a^	206.73 ± 2.82^d^
β-Sitosterol	1,709.09 ± 13.14^c^	32.10 ± 1.17^d^	1,682.67 ± 30.49^c^	6,002.10 ± 16.05^b^	6,413.05 ± 19.25^a^
5-Avenasterol	124.60 ± 13.00^c^	ND^d^	176.72 ± 13.92^b^	1,301.81 ± 6.19^a^	127.25 ± 12.61^c^
7-Stigmastanol	ND^d^	ND^d^	208.03 ± 8.80^c^	808.20 ± 3.67^b^	2,579.17 ± 20.92^a^
β-Carotene	ND^b^	ND^b^	ND^b^	1.96 ± 0.12^b^	226.17 ± 8.68^a^

*Different letters (a, b, c, d, and e) indicate significant differences (*P* < 0.05). ND, not detected.

^¶^Trans FA includes trans-18:1, trans-18:2, and trans-18:3, which are not counted in SFA, MUFA, or PUFA.

^#^n-6/n-3 is represented by the ratio of C18:2n-6 to C18:3n-3.

The compositions of micronutrients including tocopherols, phytosterols, and β-carotene are also summarized in Table 1. The total tocopherols of SBSO and SBPO are 2,806.33 ± 14.22 mg/kg and 1,834.67 ± 91.82 mg/kg, respectively, which are higher than SO and PO at 1,230.00 ± 5.29 mg/kg and 309.33 ± 6.51 mg/kg, respectively, and negligible for LO. Both SBSO and SBPO retained more than 1,000 mg/kg of α-tocopherol with SBPO having a slightly higher amount at 1,621.23 ± 4.69 mg/kg. Whereas, SBSO contains 1,279.90 ± 8.42 mg/kg of γ-tocopherol and 277.00 ± 3.96 mg/kg of δ-tocopherol while SBPO has little of them, in accordance with previous reports (15, 20).

Sea buckthorn seed oil and SBPO are good sources of phytosterols as well (15). The total phytosterols of SBSO and SBPO in this study are 9,809.67 ± 31.68 mg/kg and 11,233.66 ± 62.99 mg/kg, respectively, much higher than SO, PO, and LO at 3,508.32 ± 28.59 mg/kg, 2,953.05 ± 50.27 mg/kg, and negligible, respectively. β-sitosterol is the dominating phytosterol in sea buckthorn, contributing to more than 6,000 mg/kg of phytosterols in both SBSO and SBPO, also in accordance with previous reports (19). Notably, SBSO contains 1,301.81 ± 6.19 mg/kg of 5-avenasterol, while SBPO has 2,579.17 ± 20.92 mg/kg of 7-stigmastanol in their phytosterol profiles. Campesterol is also present in SBSO and SBPO at 656.57 ± 0.94 mg/kg and 206.73 ± 2.82 mg/kg, respectively.

β-Carotene was also evaluated in this study since it was reported to be the most abundant carotenoid of sea buckthorn berries (15). It was found that β-carotene was relatively low at 226.17 ± 8.68 mg/kg in SBPO and at a trace level in SBSO, and not detected in SO, PO, and LO.

### Daily energy intake, body weights, and relative organ weights

**FIGURE 1:**
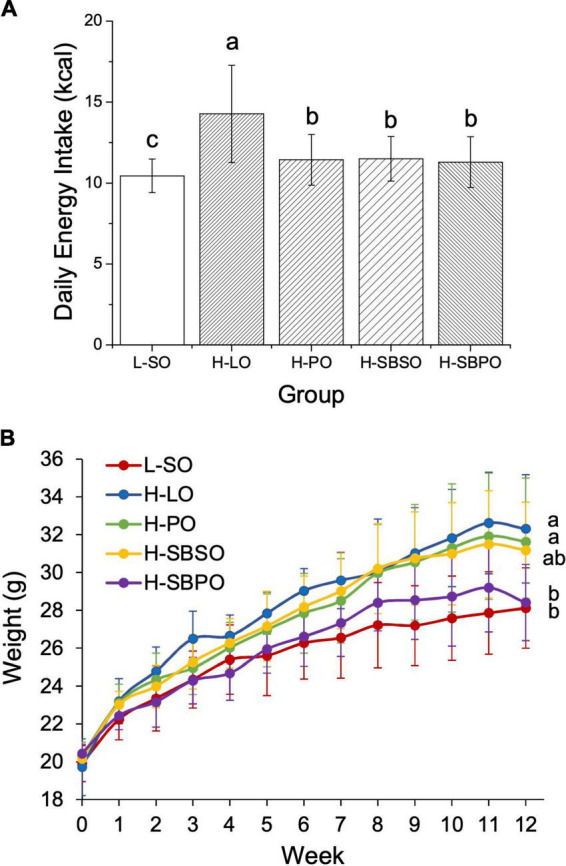
**(A)** Daily energy intake, **(B)** body weight change over a 12-week treatment period fed with one of the five diets (*n* = 12). L-SO, low-fat diet with soybean oil; H-LO, high-fat diet with lard oil; H-PO, high-fat diet with peanut oil; H-SBSO, high-fat diet with sea buckthorn seed oil; H-SBPO, high-fat diet with sea buckthorn pulp oil. Different letters (a, b, and c) indicate significant differences (*P* < 0.05) in mice’s daily energy intake and final body weight between each other.

Mice had access to feed and water *ad libitum* over the 12-week treatment period, to assure adequate food, energy, and nutrient intake on both low-fat diet and high-fat diets blended with different oils, and also to assess if any of the diets would result in anorexia or over-eating. Daily energy intakes are comparable among H-PO, H-SBSO, and H-SBPO, which are slightly higher than that of L-SO and much lower than that of H-LO (Figure 1A). Considering that the four HFDs were designed using the same formula and only differed in oil selection (Supplementary Table 1), the higher daily intake of H-LO was probably due to the distinctive taste of animal fat compared to plant oil. Here, L-SO is benchmarked as a “healthy dietary pattern” reference with low-fat content and slightly lower daily energy intake, while H-LO is regarded as an “unhealthy dietary pattern” reference due to the higher daily energy intake, in comparison to the other three. The following good/bad judgments are made among the three parallel HFD groups, H-PO, H-SBSO, and H-SBPO.

The initial weights of the five groups are all the same at 0 week (Figure 1B). L-SO, as the only low-fat diet, led to the lowest final body weight at 12 weeks (Figure 1B). H-LO had a significantly higher final body weight than L-SO and H-SBPO, which was in accordance with the highest daily energy intake of H-LO among all groups (Figure 1). H-SBPO had a similar final body weight to L-SO, and significantly lower final body weight compared to H-PO when daily energy intakes were similar in both H-SBPO and H-PO (Figure 1B). This implied that a high-fat diet comprised of SBPO is better than that of PO for body weight control, and the former could yield analogous final body weight to low-fat diet after 12 weeks.

The relative organ weights are calculated as the proportions of the actual organ weights to body weight. In this study, the relative liver weight, relative perirenal fat weight, and relative epididymal fat weight were measured for five groups after a 12-week treatment. H-SBPO and H-SBSO groups had comparable relative liver weights to the other three groups (Supplementary Figure 1A). High-fat diet led to a significantly more accumulated relative perineral fat in H-PO and H-SBSO groups compared to a low-fat diet L-SO, but brought comparable amounts of relative perirenal fat in H-SBPO and L-SO (Supplementary Figure 1B). Similarly, a high-fat diet resulted in significantly higher levels of relative epididymal fat weight in H-PO than low-fat diet L-SO, but comparable levels of relative epididymal fat weight in H-SBPO, H-SBSO, and L-SO (Supplementary Figure 1C). Taken together, consuming SBPO is less likely to accumulate visceral fat compared to consuming PO in a high-fat diet.

### Serum and hepatic lipids

**FIGURE 2:**
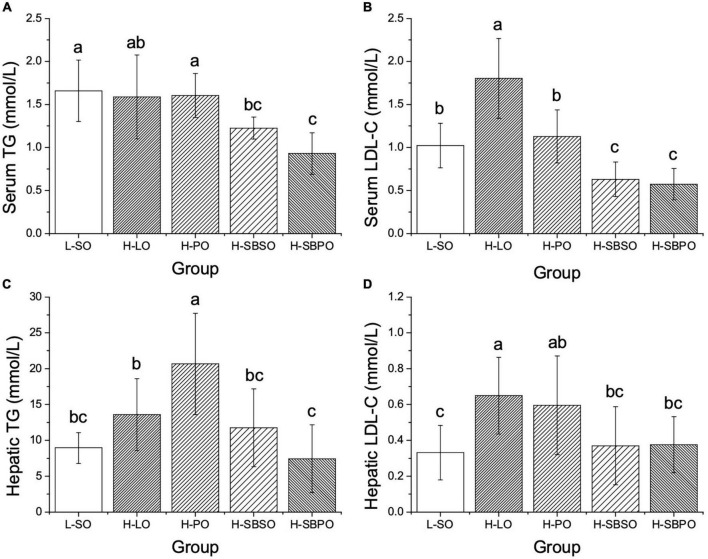
Comparisons of **(A)** serum TG, **(B)** serum LDL-C, **(C)** hepatic TG, and **(D)** hepatic LDL-C of mice after 12 weeks fed with one of the five diets (*n* = 10–12). L-SO, low-fat diet with soybean oil; H-LO, high-fat diet with lard oil; H-PO, high-fat diet with peanut oil; H-SBSO, high-fat diet with sea buckthorn seed oil; H-SBPO, high-fat diet with sea buckthorn pulp oil. Different letters (a, b, and c) indicate significant differences (*P* < 0.05) of mice serum and hepatic TG and LDL-C between each other.

Serum and hepatic TG, TC, LDL-C, and HDL-C were examined among five groups. In serum, it was found that both H-SBPO and H-SBSO resulted in lower serum TG than H-PO and L-SO, with H-SBPO having lower serum TG than H-LO (Figure 2A). Besides, both H-SBPO and H-SBSO yielded lower serum LDL-C than H-PO, H-LO, and L-SO (Figure 2B). Moreover, both H-SBPO and H-SBSO showed slightly lower serum TC than H-LO (Supplementary Figure 2A) but also lower serum HDL-C than the other three groups (Supplementary Figure 2B).

Likewise, in the liver, both H-SBPO and H-SBSO led to lower hepatic TG than H-PO, with H-SBPO resulting in lower hepatic TG than H-LO (Figure 2C). Furthermore, both H-SBPO and H-SBSO yielded lower hepatic LDL-C than H-LO (Figure 2D). However, no significant difference of hepatic TC was observed among H-SBPO, H-SBSO, H-PO, and H-LO (Supplementary Figure 2C). Also, both H-SBPO and H-SBSO presented lower hepatic HDL-C than H-PO and comparable levels to H-LO (Supplementary Figure 2D).

To summarize, SBPO and SBSO improved blood and hepatic lipid profiles primarily by suppressing serum TG, LDL-C, and TC, as well as hepatic TG and LDL-C.

### Liver pathological observation

**FIGURE 3:**
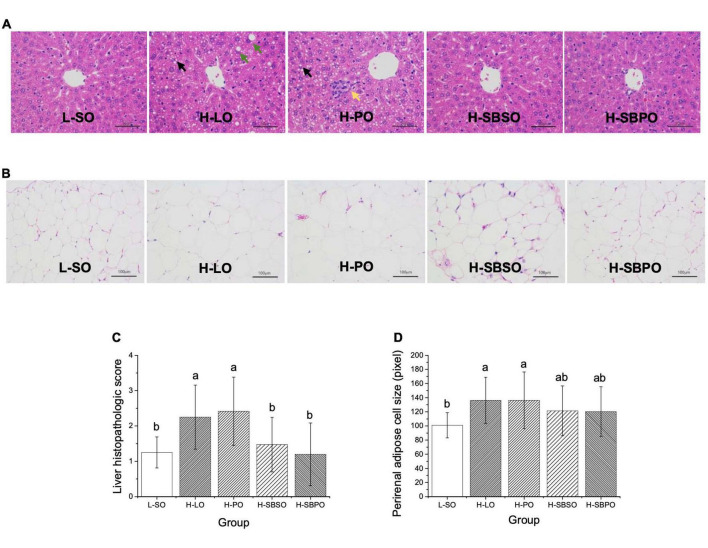
Representative images of H&E staining of **(A)** liver (enlarged 400×), **(B)** perirenal fat (enlarged 200×), **(C)** liver histopathologic scores, and **(D)** perirenal adipose cell sizes of mice treated with L-SO, H-LO, H-PO, H-SBSO, or H-SBPO (*n* = 12). L-SO, low-fat diet with soybean oil; H-LO, high-fat diet with lard oil; H-PO, high-fat diet with peanut oil; H-SBSO, high-fat diet with sea buckthorn seed oil; H-SBPO, high-fat diet with sea buckthorn pulp oil. Black arrows indicate various sizes of circular vacuoles detected in the cytoplasm; green arrows indicate the fusion of large lipid droplets in the cytoplasm; while the yellow arrow indicates the focal infiltration of lymphocytes. Different letters (a and b) indicate significant differences (*P* < 0.05) in mice liver pathological score and perirenal adipose cell size between each other.

The representative images of liver histopathologic lesions of L-SO, H-LO, H-PO, H-SBSO, and H-SBPO are presented in Figure 3A, respectively. In L-SO, hepatocyte morphology and lobular morphology were intact, nucleus size was normal, hepatocytes were mildly steatosis, and various sizes of circular vacuoles were seen in the cytoplasm (Figure 3A). In H-LO, partial hepatocyte steatosis was found in liver tissues, and various sizes of circular vacuoles were detected in the cytoplasm accompanied by fusion of large lipid droplets (Figure 3A). In H-PO, massive hepatocyte steatosis was seen in liver tissues, various sizes of circular vacuoles were observed in the cytoplasm, and focal infiltration of lymphocytes was found in some regions (Figure 3A). H-SBSO showed similar liver histopathology to L-SO with intact hepatocyte morphology and lobular morphology, normal nucleus size, mild hepatocyte steatosis, and various sizes of circular vacuoles in the cytoplasm (Figure 3A). H-SBPO also exhibited intact hepatocyte morphology and lobular morphology, normal nucleus size, and mild hepatocyte steatosis, comparable to L-SO and H-SBSO (Figure 3A). Based on the aforementioned observations, H-SBPO and H-SBSO were scored analogous to L-SO, but scored significantly lower than H-LO and H-PO for liver steatosis (Figure 3C), suggesting that SBPO and SBSO showed better effect than PO on alleviating hepatic cell damage caused by HFD.

### Perirenal adipose cell size

Adipose cell size could reflect the lipid accumulation level under the same magnification of the microscope (24). In this study, the average diameter (pixel) on the image was used to represent the size of perirenal adipose cells. The representative images of perirenal fat H&E staining of L-SO, H-LO, H-PO, H-SBSO, and H-SBPO are shown in Figure 3B, respectively. H-LO and H-PO had significantly larger perirenal adipose cells than L-SO, while H-SBSO and H-SBPO only had slightly larger perirenal adipose cells than L-SO when no significant difference was identified among these three groups (Figure 3D), indicating that SBPO and SBSO did a better job than PO in reducing perirenal fat accumulation caused by HFD.

### Fecal microbial diversity

**FIGURE 4:**
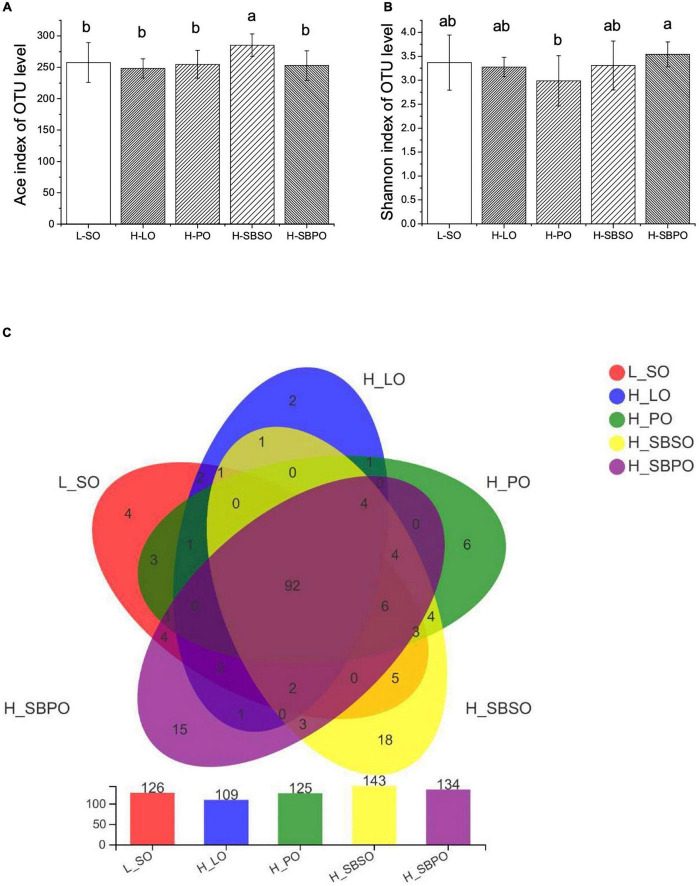
α-Diversity of gut microbiota illustrated by **(A)** Ace index, **(B)** Shannon index, and **(C)** Venn diagram of gut microbiota at OTU level (*n* = 11–12). L-SO, low-fat diet with soybean oil; H-LO, high-fat diet with lard oil; H-PO, high-fat diet with peanut oil; H-SBSO, high-fat diet with sea buckthorn seed oil; H-SBPO, high-fat diet with sea buckthorn pulp oil. Different letters (a and b) indicate significant differences (*P* < 0.05) of mice gut microbiota α-diversity (reflected by Ace and Shannon index) between each other.

The richness and diversity of fecal microbial communities are illustrated by α-diversity via Ace and Shannon index, respectively (Figure 4). At 97% identity of OTUs, H-SBSO significantly increases microbial community richness compared to the other four diets (Figure 4A). While H-SBPO is correlated with a more diverse microbial community than H-PO (Figure 4B).

Venn diagrams further showed that five groups shared 92 OTUs, and H-SBSO and H-SBPO have the most abundant unique OTUs (18 and 15 OTUs, respectively), demonstrating that H-SBSO and H-SBPO had more diversified microbial composition at the OTU level than the other three groups (Figure 4C).

β-diversity elucidated by principal coordinates analysis (PCoA) reflects the distance between groups based on their overall similarities (Supplementary Figure 3). It was shown that the majority of variations of microbial community could be explained by PC1 (25.77%) and further clarified by PC2 (13.21%) (Supplementary Figure 3). As expected, H-SBSO was closely clustered with L-SO (Supplementary Figure 3). No overlaps were found between H-LO and L-SO, H-PO, and H-SBSO (Supplementary Figure 3). A small overlap was found between H-LO and H-SBPO (Supplementary Figure 3), which may be due to the similarity of their SFA/MUFA/PUFA ratio (Table 1).

### Fecal microbial composition and relative abundance

**FIGURE 5:**
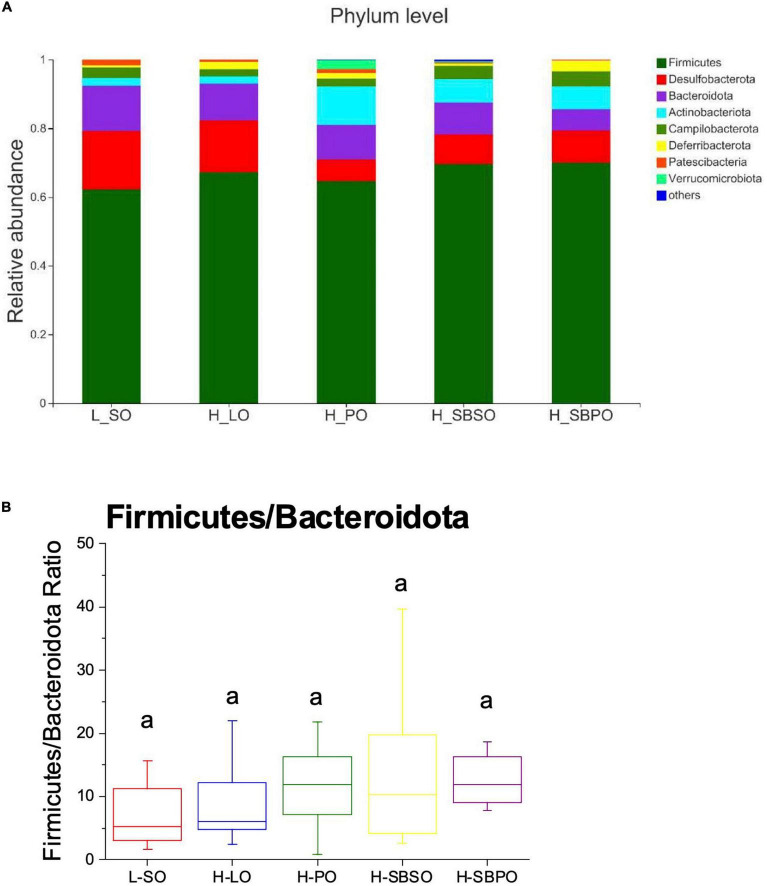
**(A)** Bacterial taxonomic profile (>0.01%) of gut microbiota at phylum level. **(B)** The ratio of Firmicutes to Bacteroidota at the phylum level (*n* = 11–12). L-SO, low-fat diet with soybean oil; H-LO, high-fat diet with lard oil; H-PO, high-fat diet with peanut oil; H-SBSO, high-fat diet with sea buckthorn seed oil; H-SBPO, high-fat diet with sea buckthorn pulp oil. The same letter a indicates no significant difference of Firmicutes to Bacteroidota ratio observed between each other (*P* < 0.05).

At phylum level, eight phyla were identified taxonomically among five groups including *Firmicutes, Desulfobacterota, Bacteroidota (Bacteroidetes), Actinobacteria, Campilobacterota, Deferribacterota, Patescibateria, and Verrucomicrobia*, and the rest were unclassified bacteria (Figure 5A). Among them, *Firmicutes* was the most prominent phylum, accounting for 62.18% (L-SO), 67.20% (H-LO), 64.69% (H-PO), 69.58% (H-SBSO), and 69.95% (H-SBPO) of the total identified bacteria, respectively (Figure 5A). The relative abundance of Firmicutes and ratio of Firmicutes to *Bacteroidota* (F/B) was comparable among five groups (Figure 5B).

**FIGURE 6:**
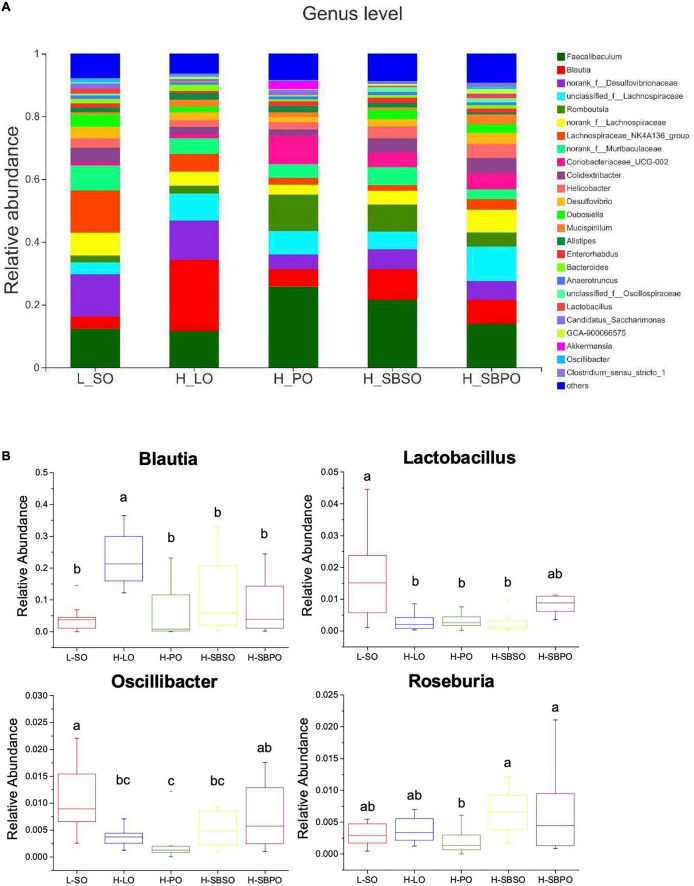
**(A)** Bacterial taxonomic profile (>0.01%) of gut microbiota at genus level. **(B)** The relative abundance of *Blautia*, *Lactobacillus*, *Oscillibacter*, and *Roseburia* at the genus level (*n* = 11–12). L-SO, low-fat diet with soybean oil; H-LO, high-fat diet with lard oil; H-PO, high-fat diet with peanut oil; H-SBSO, high-fat diet with sea buckthorn seed oil; H-SBPO, high-fat diet with sea buckthorn pulp oil. Different letters (a, b, and c) indicate significant differences (*P* < 0.05) in the relative abundance of *Blautia*, *Lactobacillus*, *Oscillibacter*, and *Roseburia* between each other.

At the genus level, *Faecalibaculum*, *Blautia*, *norank_f_Desulfovibrionaceae*, *unclassified_f_Lachnospiraceae*, and *Romboutsia* dominated the genus profiles of five groups, and along with *Lactobacillus*, *Oscillibacter*, and *Roseburia* appeared in the top 30 of genera based on relative abundance (Figure 6A). Among the top 30 abundant genera, 21 of them showed difference between groups (Supplementary Figure 4). Specifically, *Blautia* was significantly boosted by the H-LO diet (Figure 6B). The relative abundance of *Lactobacillus* was significantly lower in H-LO, H-PO, and H-SBSO, while was at a comparable level in H-SBPO, compared to L-SO (Figure 6B). Moreover, *Oscillibacter* was more abundant in H-SBPO than in H-PO (Figure 6B). Likewise, *Roseburia* was richer in H-SBPO and H-SBSO than H-PO (Figure 6B). The distinctive findings at genus level were in accordance with the more diverse microbial community of H-SBPO than others (Figure 4B).

## Discussion

To the best of our knowledge, this is the first study to reveal the influence of SBPO on the gut–liver axis and to determine the differences between SBSO and SBPO in terms of bioactive compound profiles and health effects on metabolic disorders along with gut microbiota dysbiosis. By switching the fat content of HFD from PO to SBSO or SBPO, the present study demonstrates that SBSO and SBPO have the potential to ameliorate HFD-induced lipid metabolism disorders including obesity, dyslipidemia, and liver steatosis, and meanwhile, modulate gut microbiota in C57BL/6J mice. Specifically, SBSO and SBPO reduced visceral fat accumulation and adipose cell size, suppressed serum TG, TC, and LDL-C levels, decreased hepatic TG and LDL-C levels, lowered hepatic cell damage score, and altered gut microbiota by enhancing the richness and diversity of microbiota community.

Although coming from the same plant, SBSO and SBPO differ in bioactive compound profiles mainly due to sea buckthorn’s divergent metabolism but not the manufacturing processes for oil extraction (15, 20). Our study shows that SBPO comprises about 31% of palmitoleic acid C16:1n-7, and contains slightly higher levels of α-tocopherol and β-sitosterol and much higher 7-stigmastanol and β-carotene than SBSO (Table 1). While SBSO exhibits a more balanced n-6/n-3 ratio close to 1.3:1 in fatty acid composition, it contains notably larger quantities of γ-tocopherol, campesterol, and 5-avenasterol than SBPO (Table 1).

The varied effects of SBSO and SBPO in relieving metabolic disorders can be partly attributed to their difference in bioactive fatty acids and other compounds. Sea buckthorn fruit oil extract featured with 81% of concentrated palmitoleic acid was able to reduce body weight, visceral fat accumulation, and adipose cell size, improve blood lipid indicators, and alleviate liver cell damage in HFD-induced hyperlipidemia hamsters, possibly through the AMPK and Akt pathways (24). Particularly, palmitoleic acid itself was regarded as the potential nutraceutical to modulate NAFLD, since it showed the capacity to alleviate liver steatosis and inflammation in HFD-fed mice (34, 35). Our results are in accordance with the previous study (24), showing that SBPO is superior to PO in ameliorating obesity, decreasing visceral fat weight and size, improving serum and hepatic lipid profiles, and preventing liver steatosis in mice on HFD (Figures 1–3 and Supplementary Figures 1, 2), likely due to the presence of palmitoleic acid in SBPO (Table 1) (34, 35). Dietary SBSO was shown to have hepatoprotective properties via reducing blood triglyceride and cholesterol as well as liver lipid peroxidation (19, 25, 28). The present study shows consistent results that SBSO exhibits similar or weaker effects in alleviating HFD-induced liver steatosis and related metabolic disorders than SBPO, but is better than PO when present at similar dietary concentrations (Figures 1–3 and Supplementary Figures 1, 2). High concentrations of various tocopherols and phytosterols in SBSO similar to SBPO, and the more balanced n-6/n-3 ratio of SBSO than PO may account for the observed health effects (Table 1). α-tocopherol high in SBPO and SBSO is commonly regarded as an antioxidant that relieves oxidative stress preventing the transition from simple steatosis to NASH (36). As the predominant phytosterols in SBPO and SBSO, β-sitosterol, campesterol, and stigmasterol were proven to have positive effects in reducing serum cholesterol, LDL-C, and prevention of NAFLD (37); additionally, β-sitosterol could also play an important role in alleviating NAFLD by decreasing hepatic cholesterol in mice on high-fat Western diet (38). Moreover, a balanced n-6/n-3 ratio close to 1:1 is suggested to maintain health and prevent the development of chronic diseases, such as NAFLD and obesity (39, 40). It is worth noting that, in this study, dietary SBSO showed similar but slightly weaker effects than SBPO in alleviating HFD-induced liver steatosis and dyslipidemia, while they yielded differences in the richness and diversity of gut microbiota, especially at the genus level (Figures 4, 6). We speculate that the similarities in relieving liver steatosis-associated metabolic disorders are attributed to the comparable phytosterol and tocopherol compositions (19), and the differences in gut microbiota could be due to the different fatty acid profiles and carotenoid contents, as SBPO is featured with high palmitoleic acid and β-carotene while SBSO has a more balanced n-6/n-3 ratio (20), though further research investigating their specific correlations is needed. Taken together, the health effects of SBPO and SBSO as diet interventions for liver steatosis and related metabolic disorders may well be some synergic functions of lipophilic bioactive compounds including fatty acids, tocopherols, phytosterols, and carotenoids.

Furthermore, the present study reveals that SBSO and SBPO dietary intervention would also alter gut microbiota composition suggesting that their targeted receptors are in the gut–liver axis. Particularly, SBSO increased the richness of the microbial community while H-SBPO increased the diversity of the microbial community compared to PO (Figure 4B); both of them had a higher abundance of unique OTUs than others, again verifying the increased diversity of microbial community caused by SBSO and SBPO (Figure 4C). These findings are not consistent with a previous study, in which SBSO did not change the richness and diversity of the microbial community in hypercholesterolemia hamsters (25). This may be attributable to the dosage difference of SBSO between the two studies. Besides, the present study did not observe the difference in F/B ratio at the phylum level in C57BL/6J mice (Figure 5B). Increased F/B ratio is usually regarded as an indicator of obesity (41). Whereas, other researchers reported that obesity and diabetes were not correlated with the F/B ratio in C57BL/6J mice (42). In addition, at the genus level, SBPO is correlated with a higher abundance of *Lactobacillus, Roseburia*, and *Oscillibacter*, and SBSO with a higher abundance of *Roseburia* than PO; meanwhile, higher daily energy intake for LO is correlated with a significantly higher level of *Blautia* in mice fecal microbiota than the others in the present study (Figure 6B). *Lactobacillus* belonging to the largest phylum *Firmicutes* is commonly used as probiotics (8). It was observed that patients with NASH decreased intrahepatic TG levels once consuming probiotic formula containing *Lactobacillus* and *Bifidobacterium* (8). Supplementations of fish oil suppressed hepatic TLR4 expression and white adipose tissue inflammation in concurrence with an increased level of *Lactobacillus* (8). *Roseburia* also belongs to the *Firmicutes* phylum. Reduced abundance of *Roseburia* was observed in the obese subject compared to lean ones (43), and *Roseburia* supplementation ameliorated liver steatosis and inflammation in murine models (44). *Oscillibacter* was found significantly lower in patients with NAFLD compared to healthy ones (45) and was believed to exhibit an important function in the prevention of NAFLD (46). *Blautia* genera were enriched in both NASH and NAFLD groups compared to the healthy control (47). Although our observations are in accordance with the aforementioned studies, the presence of specific genera in metabolic disorders is controversial in most cases so their contributions to liver steatosis are still under debate.

Current observations suggest that dietary oil is likely to alleviate lipid metabolism disorders by regulating gut microbiota, which is probably attributed to the existence of short-chain fatty acid (SCFA)-producing gut microbiota. SCFAs are the metabolites of indigestible carbohydrates produced by various microbiota in the large intestine and are capable to inhibit cholesterol synthesis, obesity, and inflammation (13). A previous study revealed that wild melon seed oil could reduce plasma cholesterol levels in part by boosting the production of fecal SCFAs (13). Moreover, the phosphorylation of the energy sensor AMPK and the corresponding AMPK/Akt signaling pathway could reduce the biosynthesis of glucose, cholesterol, and triacylglycerol in the liver (24). Previous research reported that sea buckthorn fruit oil extract containing 81% of concentrated palmitoleic acid showed an anti-hyperlipidemia effect and alleviated liver impairment in HFD-fed hamsters by promoting the phosphorylation of AMPK (24). However, no one has built up the bridge between SCFA produced by gut microbiota and AMPK/Akt signaling pathway with the dietary intervention of SBPO/SBSO. Although not included in the present study, the mechanism that SBPO and SBSO ameliorate lipid metabolism disorders by regulating gut microbiota is worth for further investigation.

## Conclusion

In summary, the present study reveals the health effects of SBPO on the gut–liver axis by evaluating indicators of liver steatosis and related metabolic disorders, as well as gut microbiota dysbiosis induced by HFD. Biochemicals including fatty acids, vitamin E, and phytosterols in SBPO and SBSO were determined. This study demonstrates that dietary SBPO has the potential to curb the development of lipid metabolism disorders, such as NAFLD, obesity, and dyslipidemia, by modulating gut microbiota.

## Data availability statement

The data presented in this study are deposited in the NCBI repository, accession number PRJNA894340, available at https://www.ncbi.nlm.nih.gov/bioproject/PRJNA894340.

## Ethics statement

The animal study was reviewed and approved by Shanghai Medicine Industry Research Institute (License Number, SYXK(Hu) 2014-0018).

## Author contributions

ZW and SZ executed the research and wrote the first draft of the manuscript. SZ recruited the funding. All authors formulated the research questions, designed the study, analyzed and interpreted the data, and approved the final draft of the manuscript.

## Footnotes

1
https://cloud.majorbio.com/
