# Supplementary material for: Sea buckthorn pulp and seed oils ameliorate lipid metabolism disorders and modulate gut microbiota in C57BL/6J mice on high-fat diet
Source: Front Nutr. 2022 Dec 8;9:1067813. doi: 10.3389/fnut.2022.1067813 (PMC9773879; doi:10.3389/fnut.2022.1067813)
Supplement: Supplementary file 1 [file Data_Sheet_1.docx]

Table S1 Experimental diet formulas

| **Ingredients (g/100g)** | **L-SO** | **H-LO, H-PO, H-SBSO, H-SBPO** |
| --- | --- | --- |
| Casein | 14.00 | 23.31 |
| L-Cystine | 0.18 | 0.35 |
| Corn Starch | 49.57 | 8.48 |
| Maltodextrin 10 | 12.50 | 11.65 |
| Sucrose | 10.00 | 20.14 |
| Cellulose,BW200 | 5.00 | 5.83 |
| tBHQ | 0.0008 | 0.00 |
| Tested Oils* | 4.00 | 23.60 |
| Mineral Mix S10022M | 3.50 | 0.00 |
| Mineral Mix S10026 | 0.00 | 1.17 |
| DiCalcium Phosphate | 0.00 | 1.51 |
| Calcium Carbonate | 0.00 | 0.64 |
| Potassium Citrate, 1 H2O | 0.00 | 1.92 |
| Vitamin Mix V10001 | 0.00 | 1.17 |
| Vitamin Mix V10037 | 1.00 | 0.00 |
| Choline Bitatrate | 0.25 | 0.23 |
| Protein | 14.2 | 23.7 |
| Carbohydrate | 72.1 | 41.4 |
| Fat | 4.0 | 23.6 |
| Total kcal/100g | 385.0 | 473.0 |

*Tested oil was refined soybean oil which comprised 4% (w/w) of L-SO group; besides, tested oils were lard oil, refined peanut oil, sea buckthorn seed oil and sea buckthorn pulp oil, which comprised 24% (w/w) of H-LO, H-PO, H-SBSO and H-SBPO groups, respectively.

c

b

a

b

a

ab

ab

ab

a

a

a

b

ab

a

a

b

ab

ab

Figure S1 Relative organ weight (of final body weight) of mice after 12 weeks (n=12), (a) relative liver weight, (b) relative perirenal fat and (c) relative epididymis fat. L-SO, low-fat diet with soybean oil; H-LO, high-fat diet with lard oil; H-PO, high-fat diet with peanut oil; H-SBSO, high-fat diet with sea buckthorn seed oil; and H-SBPO, high-fat diet with sea buckthorn pulp oil. Different letters (a, and b) indicate significant differences (P<0.05) of mice relative liver and visceral fat weight between each other.

Figure S2 Comparisons of (a) serum TC, (b) serum HDL-C, (c) hepatic TC and (d) hepatic HDL-C of mice after 12-weeks fed with one of the five diets (n=10-12): L-SO, low-fat diet with soybean oil; H-LO, high-fat diet with lard oil; H-PO, high-fat diet with peanut oil; H-SBSO, high-fat diet with sea buckthorn seed oil; and H-SBPO, high-fat diet with sea buckthorn pulp oil. Different letters (a, b, and c) indicate significant differences (P<0.05) of mice serum and hepatic TC and HDL-C between each other.

d

b

c

a

Figure S3 β-diversity of gut microbiota elucidated by principal co-ordinates analysis (PCoA) plot (n=11-12). L-SO, low-fat diet with soybean oil; H-LO, high-fat diet with lard oil; H-PO, high-fat diet with peanut oil; H-SBSO, high-fat diet with sea buckthorn seed oil; and H-SBPO, high-fat diet with sea buckthorn pulp oil.


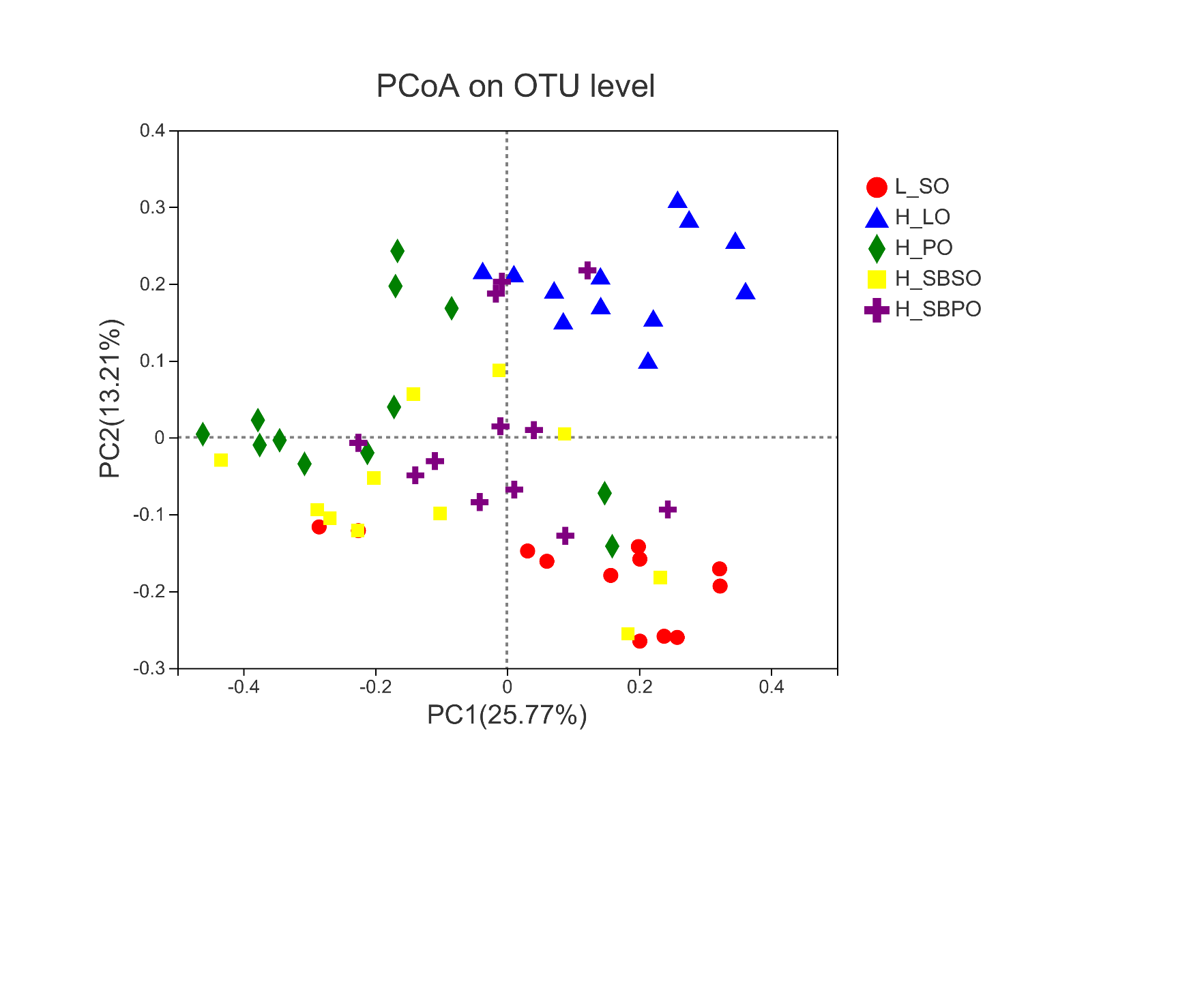


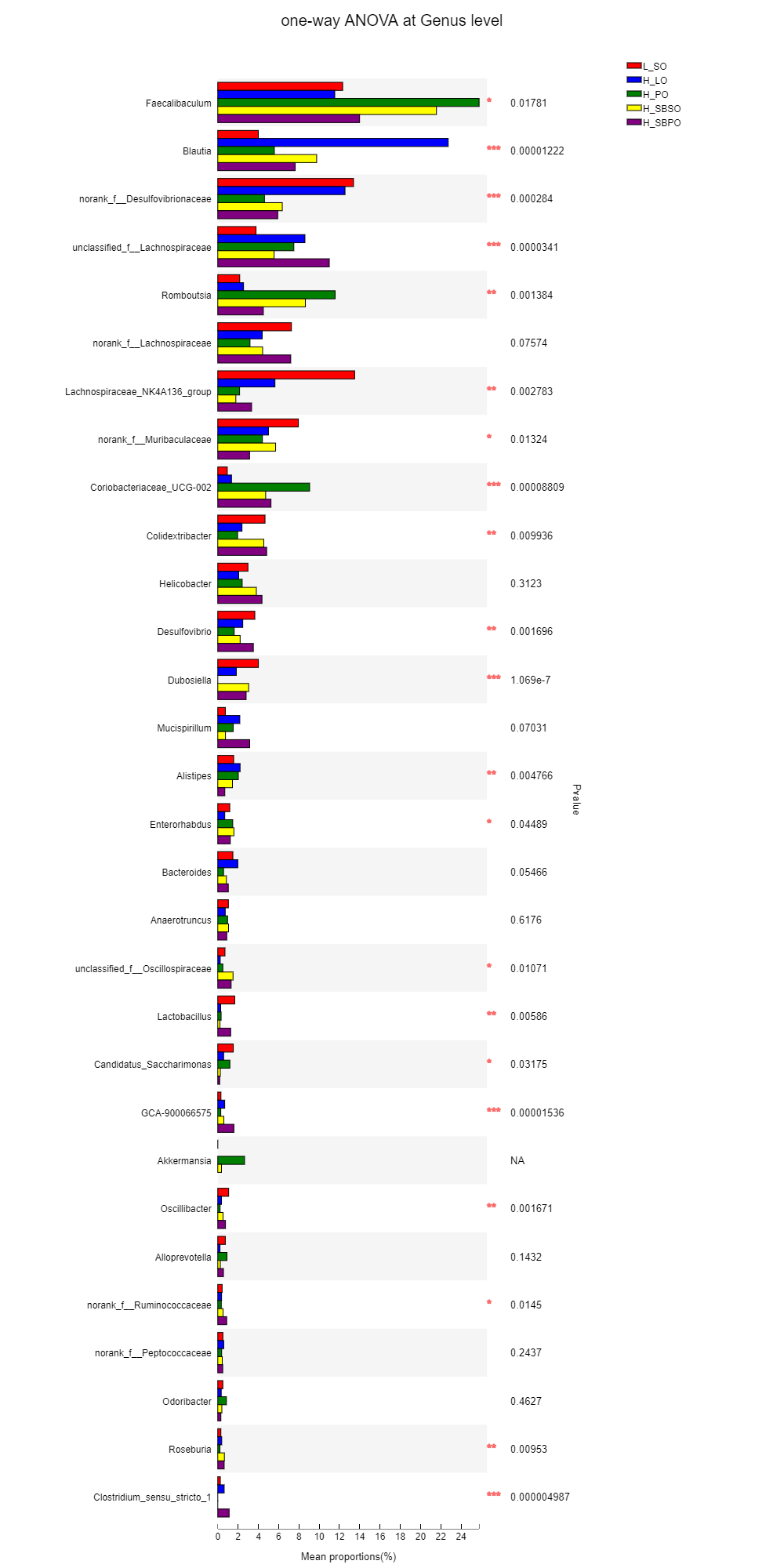


Figure S4 One-way ANOVA on the top 30 abundant genera in mice feces of five groups (n=11-12): L-SO, low-fat diet with soybean oil; H-LO, high-fat diet with lard oil; H-PO, high-fat diet with peanut oil; H-SBSO, high-fat diet with sea buckthorn seed oil; and H-SBPO, high-fat diet with sea buckthorn pulp oil. Significant differences between groups are denoted with * P<0.05, ** P<0.01, *** P<0.001.
